# (Co)variance components and genetics parameter estimation for linear traits in Holstein cattle in Indonesia: traits related to foot/leg and udder

**DOI:** 10.5194/aab-61-491-2018

**Published:** 2018-12-20

**Authors:** Agus Susanto, Suyadi, Veronica Margareta Ani Nurgiartiningsih, Luqman Hakim

**Affiliations:** 1 Graduate Program, Faculty of Animal Science, Brawijaya University (UB), Malang, Indonesia; 2 Faculty of Animal Science, University of Jenderal Soedirman (UNSOED), Purwokerto, Indonesia; 3 Faculty of Animal Science, Brawijaya University (UB), Malang, Indonesia

## Abstract

The availability of (co)variance components and genetics parameter estimates for traits included in a selection program is crucial since the estimated breeding values of the selected traits are computed based on the available (co)variance components and genetics parameters. The present study aimed to estimate (co)variance components and genetics parameters for linear traits related to foot/leg and udder (i.e. rear legs set, foot angle, udder depth, and teat length) in Holstein cattle in Indonesia. Linear traits were measured (instead of scored) on 310 lactating Holstein cows raised in the National Breeding Centre for Dairy Cattle and Forage of Indonesia (BBPTUHPT Baturraden). These were nearly all cows in lactation owned by the centre at the time of study. Lactating cows which were not measured during study were those which are technically difficult to handle. The Average Information Restricted Maximum Likelihood (AI-REML) method of the DMU program was used to estimate the (co)variances and genetics parameters of the considered linear traits. A four-multivariate animal model was employed by including farm (fixed), animal (random), and age (covariate) effects in the model of analysis. The phenotypic means (standard deviation) for rear legs set, foot angle, udder depth, and teat length were 139.70 (6.03), 50.65 (5.04), 10.67 (6.19), and 5.27 (0.96), respectively. The results showed that the estimated heritability ($h^{2}$) was 0.334, 0.236, 0.147, and 0.213 for rear legs set, foot angle, udder depth, and teat length, respectively. The genetic (phenotypic) correlations between linear traits rear legs set–foot angle, rear legs set–udder depth, rear legs set–teat length, foot angle–udder depth, foot angle–teat length, and udder depth–teat length were -0.08 (-0.043), -0.6 (0.002), 0.101 (0.036), 0.002 (-0.017), -0.186 (-0.146), and -0.834 (0.019), respectively. The present study concluded that the linear traits could be used in the selection program, though the traits should be properly weighted to avoid deteriorating selection response.

## 1 Introduction

Since its establishment in the 1950s, the National Breeding Centre for Dairy Cattle and Forage (known as BBPTUHPT Baturraden) of Indonesia has played a major role in providing dairy cows to domestic farmers through its genetics improvement program. BBPTUHPT Baturraden, which is the main government institution for genetics improvement of Indonesian dairy cattle, serves the country to rear, produce, and improve genetically dairy cattle, specifically Holstein cattle. The centre has focused its genetics improvement program on milk yield and its component traits. Inclusion of linear traits or other traits in its genetics improvement program was not considered until recently.

Milk yield and its components of dairy cows in general have been successfully increased through both genetics and management. Milk yield per lactation has increased dramatically in the last 40 years and some cows were reported to be able to produce more than 20 000 kg per lactation (Oltenacu and Broom, 2010). The milk yield increase of 5997 kg from 1957 to 2007 is attributed mostly (56 %) to genetics improvement (VanRaden, 2004). Many studies have shown that selecting dairy cows focusing merely on milk yield (and its components) and ignoring other traits, especially linear traits, has resulted in declined performances in animals' health and reproduction performances despite the increased production traits.

Rauw et al. (1998) reviewed quite extensively the opposite relationship between high-production cows and health performance. The different direction relationship between high-production cows and health expenses has been reported from a classical selection study (Jones et al., 1994). Ingvartsen et al. (2003) reported the evidence of genetics correlation between milk yield and the incidence of ketosis, ovarian cyst, mastitis, and lameness. Health performances also experienced a similar trend along with the successful increase in milk production due to the selection program. The relationship between milk yield and risk of infection of mastitis is clear and if the animals continue to be selected for high milk yield, the situation will worsen (Ingvartsen et al., 2003). Milk yield of previous lactation is associated with retained placenta, mastitis, and milk fever (Fleischer et al., 2001).

Lucy (2001) has shown the negative association between the successful increase in milk yield and reproductive performances over the last decades. Similar to what has happened in the US, first conception rate has decreased in Ireland (Roche et al., 2000) and the United Kingdom (Royal et al., 2000). Despite an increased milk yield from 7800 kg to 10 200 kg within the period of 1991–2000, pregnancy rate and cyclicity have declined by up to 6 % and 7.6 %, respectively, and incidence of inactive ovaries increased by up to 8 % (López-Gatius, 2003). This phenomenon was addressed to the presence of undesirable genetic correlation between milk yield and fertility traits (Pryce et al., 2004) or due to both management and the negative genetic correlation between those traits (Lucy, 2007).

Linear traits are correlated with some economically important traits in livestock and, to some extent, the correlation is genetic. Selecting animals based upon a particular trait will affect the mean of the correlated traits. This is critical especially when the genetics correlation is in the opposite direction, since the improvement in one trait will decrease the performance of the other traits. The International Committee on Animal Recording (ICAR, 2011) has recommended recording linear traits on a breeding program since the genetics correlation is present between linear traits and production traits.

Selection of traits that need to be recorded in a genetics improvement program needs to be done because it is inefficient and expensive to record all traits included in the breeding objective. Genetics improvement of a trait can be achieved by including traits that are genetically correlated and easier/cheaper to record in the selection program. Therefore the information about the (co)variance components and genetics parameters of alternative traits of a population is important. (Co)variance components and parameters genetics of linear traits of Indonesian Holstein cattle, to the best of our knowledge, have not been reported. Thus the objective of this present paper was to estimate the necessary genetics parameters of linear traits for Holstein cows in Indonesia.

## 2 Material and methods

### 2.1 Data

Linear traits related to foot/leg and udder, including rear legs set, foot angle, udder depth, and teat length, were reported in the study. The corresponding linear traits of Holstein cattle in Indonesia were measured during the months of July and August 2017 at the National Breeding Centre for Dairy Cattle and Forage of Baturraden, Indonesia. As many as 310 lactating dairy cows were measured for their linear traits. The udder depth trait was measured about an hour before milking in the afternoon; animals are milked twice daily: early morning and afternoon. All animals were reared in two separated farms, namely Tegalsari and Limpakuwus farms. Animal age and pedigree data were obtained from the database available in BBPTUHPT Baturraden. Animals with an unknown date of birth were assigned as data missing for the age variable and unknown parents were also assigned as missing. All animals are reared under an intensive system in a free stall barn where grass and concentrate are provided twice daily and water is provided ad libitum.

Measuring linear traits has not been initiated so far in the Indonesian National Breeding Centre for Dairy Cattle and Forage, though in this project 10 linear traits related to conformation, foot and leg, and udder were introduced. However, in this paper only four linear traits are reported, i.e. rear legs set, foot angle, udder depth, and teat length. The linear traits were measured directly on animals as opposed to being scored/classified according to the World Holstein Friesian Federation (WHFF, 2005) definition. Lack of certified classifiers for typing/scoring for these linear traits inspired the author to measure the traits instead of scoring them. Rear legs set, foot angles, and teat length were measured for both left and right parts. Rear legs set was measured with the digital angle level of Nankai^®^. Foot angle was measured with a conventional angular measure; teat length was measured with a standard tape measure; udder depth was measured with a calliper. Rear legs set and foot angle were both measured in angle degree unit (${}^{\circ }$), whereas udder depth and teat length were measured in centimetre unit (cm). Animal pedigree data required to compose the numerator relationship matrix (A) of additive genetics of animals were collected from the database at BBPTUHPT Baturraden and are available in spreadsheet files. The population structure of the data under study is presented in Table 1.

**Table 1:** Description of the dataset.

Item	Count
Number of animals measured	310
Number of animals with missing age	11
Number of animals in pedigree file	6963
Number of animals with unknown sire	305
Number of animals with unknown dam	709
Number of animals with unknown sire and dam	1931
Number of animals in pedigree file with	
unknown year of birth	3008

### 2.2 Statistical analysis

The model used in the (co)variance analysis included farm (fixed), animal genetics additive (random), and animal age (random-fixed covariate) effects. Multivariate genetics analysis including four traits at a time was chosen by employing the equal design model for all traits being analysed. The mathematical model can be written as in Eq. (1):

1 $$y_{ij}=\mu +F_{i}+b\left(X_{ij}\right)+a_{i}+e_{ij},$$

where $y_{ij}$ is the corresponding trait being analysed (rear legs set, RLS, foot angle, FA, udder depth, UD and teat length, TL), μ is the overall mean, $F_{i}$ is the fixed effect of farm, b is the regression coefficient of $y_{ij}$ on age, a is the random additive genetic effect of animals, and e includes the random residual effects. The model assumes that the variance structures for random additive genetic and residual effects are defined as var$(a)=A\sigma_{a}^{2}$ and var$\left(e\right)=I\sigma_{e}^{2}$, where A is the numerator relationship matrix and I is the identity matrix, respectively. The model also assumes no covariance between random additive genetic and residual effects (cov$\left(a,e\right)=\text{cov}\left(a,e\right)=0)$. (Co)variance estimation analysis was performed with the animal model by Average Information Restricted Maximum Likelihood (AI-REML) using the DMU software which provides the standard error of the estimates (Madsen and Jensen, 2013). The maximization likelihood computation set in running the DMU program was sparse computation of the average information method with step halving if the updated estimates were not within the parameter space (option 4). The maximization process and convergence criteria were controlled by parameters set by the default of the DMU software. Starting values (prior) for (co)variance components used the default values provided by the DMU software as well where an identity matrix is assumed for all (co)variance matrices included in the model. Since the pedigree data are not readily available to be sorted based on year of birth or generation, the inverse of the numerator relationship matrix ($A^{-1}$) was instead computed by ignoring the inbreeding coefficient (option 2). Heritability of each trait was computed following Eq. (2):

2 $$h^{2}=\frac{\sigma_{a}^{2}}{\sigma_{a}^{2}+\sigma_{e}^{2}},$$

where $\sigma_{a}^{2}$ is variance of additive genetics of animals, $\sigma_{e}^{2}$ is residual variance, and $h^{2}$ is heritability.

The standard errors of heritabilities and correlations between random effects including genetic correlations are computed based upon a Taylor series approximation (Madsen and Jensen, 2013).

**Table 2:** Phenotypic and standard deviation of the dataset${}^{\text{a}}$.

Linear traits	Mean	SD${}^{\text{b}}$	CV${}^{\text{c}}$
Rear legs set (${}^{\circ }$)	139.70 (6.68)	6.03 (37.53)	4.32
Foot angle (${}^{\circ }$)	50.65 (5.75)	5.04 (-0.33)	9.95
Udder depth (cm)	10.67 (5.56)	6.19 (4.06)	58.01
Teat length (cm)	5.27 (5.27)	0.96 (0.96)	18.22

${}^{\text{a}}$ Values in the brackets are computed by converting the observed values into the 1–9 scale scoring system of the World Holstein Friesian Federation (WHFF, 2005). ${}^{\text{b}}$ SD: phenotypic standard deviation. ${}^{\text{c}}$ CV: phenotypic coefficient of variation (%).

## 3 Results and discussion

It is quite a challenge to have reliable estimates of heritability and (co)variance components in developing countries since the pedigree data of the population are not well documented. Pedigree information as the basis of the variance structure of additive genetics of the animals is critical. The DMU software allows the formation of a numerator relationship matrix inverse without considering the inbreeding coefficient (option 2). Selecting option 2 in DMU will command the program to create an approximation of the true inverse of the additive relationship matrix. Phenotypic means and standard deviation of the RLS, FA, UD, and TL are presented in Table 2. In the present study, linear traits were measured instead of scored/classified, as opposed to most studies reported in the literature, for instance Toghiani Pozveh et al. (2009), Mazza et al. (2015), Wiggans et al. (2006), and Zink et al. (2012), and therefore comparing directly estimated parameters of the present study with those in the literature is not possible except for the ratio between estimates such as correlations and heritabilities. Comparing the estimated parameters with other studies was done cautiously after the estimated parameters obtained were converted into values of the 1–9 scale scoring system. However, phenotypic means of the observed traits were compared with the 1–9 scale scoring system to get a general overview of the population parameters under study compared to other studies.

Table 2 shows that the phenotypic means of the observed linear traits are closed to the intermediate score in a 1–9 scale scoring system according to the reference scale of the World Holstein Friesian Federation (WHFF, 2005). The intermediate scores of RLS, FA, UD, and TL according to the WHFF (2005) are 4–6, 4–6, 5, and 4–6, respectively. This indicates that the linear traits of Indonesian Holstein cows have not undergone selection. The observed phenotypic means of the present study of RLS and FA were slightly higher than those reported by Toghiani (2011), whereas UD is about the same. Toghiani (2011) reported that the phenotypic means (standard deviation) of RLS, FA, and UD scored in a 1–9 scale scoring system are 5.4 ± 1.1, 5.3 ± 1.3, and 5.6 ± 1.1, respectively.

An additive genetic coefficient of variation (CV) of 13 % has been reported by Berry et al. (2004) for udder depth in primiparous dairy cows, whereas Fernández et al. (1997) reported a coefficient of variation of 34.48 % for udder depth in dairy ewes. The extremely high phenotypic coefficient of variation obtained from the present study, especially udder depth (58.01 %), might indicate that the source of variation originating from non-genetic factors, such as the field technician who measured the variable, is dominant. Measuring udder depth of dairy cows kept in a free stall barn is quite challenging. Obtaining a large coefficient of variation of field data is possible and has been reported in the literature. For instance, Royal et al. (2002) reported a phenotypic coefficient of variation of commencement luteal activity (CLA) of 63 %. Samoré et al. (2012) reported CVs of 57 % and 62 % for somatic cell scores of Brown Swiss and Holstein Friesian cattle, respectively.

**Table 3:** Estimated genetic variances (diagonal) and covariances (below diagonal).

	RLS	FA	UD	TL
RLS	7.108			
FA	-0.447	4.411		
UD	-2.621	0.006	2.687	
TL	0.113	-0.163	-0.572	0.175

**Table 4:** Estimated phenotypic variances (diagonal) and covariances (below diagonal).

	RLS	FA	UD	TL
RLS	21.299			
FA	-0.865	18.725		
UD	0.029	-0.319	18.293	
TL	0.150	-0.571	0.073	0.820

### 3.1 Heritabilities and variances

The estimated genetic and phenotypic (co)variances of the present study are presented in Tables 3 and 4. The two tables show that (co)variances, both genetically and phenotypically, were observed in the population, and they varied in magnitude. Comparing the magnitude of (co)variances obtained in the present study with results from studies using the 1–9 scale scoring system, for instance, is irrelevant and thus avoided. The discussion is therefore emphasized on the variance ratio (i.e. heritabilities and correlations). The estimated heritabilities along with the standard errors are shown in Table 5.

**Table 5:** Estimated heritabilities and the standard errors.

Linear trait	$h^{2}$	SE ($h^{2}$)
RLS	0.334	0.181
FA	0.236	0.174
UD	0.147	0.066
TL	0.213	0.156

It is shown in Table 5 that rear legs set, foot angle, udder depth, and teat length of dairy cattle in Indonesia are heritable traits, though the magnitudes of the estimates varied. The estimated heritabilities ranged from 0.147 for TL to 0.334 for RLS. These results were different compared to those reported in the literature. For example, Pérez-Cabal and Alenda (2002) reported heritabilities for RLS, FA, and UD of 0.17, 0.11, and 0.24, respectively. Dal Zotto et al. (2007) reported heritabilities of 0.14, 0.23, and 0.32 for RLS, UD, and TL, respectively, on Brown Swiss cattle. Estimated heritabilities of 0.19, 0.16, 0.30, and 0.33 for RLS, FA, UD, and TL, respectively, were reported by Pryce et al. (2000). From a study of Holstein cattle in Brazil, Campos et al. (2015) reported heritability estimates of 0.21, 0.09, 0.25, and 0.38 for RLS, FA, UD, and TL, respectively. The heritability estimates could be different from study to study since the estimates are a function of additive genetic and non-genetic variances. The higher heritability estimate reflects the higher proportion of additive genetic variance to the total variance. Results of the present study show that the linear traits under study are heritable and a significant selection response might be obtained when included in a selection program. The standard error of all heritability estimates of the present study are slightly larger (0.066–0.181) than those reported in the literature, except for udder depth, which has a standard error for heritability of 0.066. For instance, Pryce et al. (2000), Pérez-Cabal and Alenda (2002), Dal Zotto et al. (2007), and Campos et al. (2015) reported standard errors of heritabilities of 0.01–0.06 for the corresponding linear traits using the restricted maximum likelihood (REML) method. The standard error of heritability estimates of the present study could probably be reduced by increasing sampling size of the study by measuring new lactating animals. In addition, the smaller standard error could possibly be obtained by improving the information accuracy of animals' pedigree such as animals' date of birth so that the true numerator additive relationship matrix of the animals can be computed by including the inbreeding coefficient. In the present study, the variance structure of animals' additive genetic was approximated by excluding the inbreeding coefficient by allowing the DMU software to run with option 2 (Madsen and Jensen, 2013).

**Table 6:** Estimated genetics (below diagonal) and phenotypic correlations (above diagonal).

	RLS	FA	UD	TL
RLS	1.000	-0.043	0.002	0.036
FA	-0.080	1.000	-0.017	-0.146
UD	-0.600	0.002	1.000	0.019
TL	0.101	-0.186	-0.834	1.000

### 3.2 Genetics and phenotypic correlation

RLS, FA, UD, and TL were correlated with each other both genetically and phenotypically, though the magnitude was variable, ranging from -0.834 to +0.101 (Table 6). Teat length and udder depth had the strongest genetic correlation (-0.834), whereas udder depth and foot angle had the weakest genetic correlation (0.002). All traits under study had weak phenotypic correlation (-0.146–0.036). The negative strong genetic correlations (-0.6 for RLS–UD and -0.834 for UD–TL) indicate that cows with more sickled rear legs tended to have genetically shallower udders and cows with genetically deeper udder depth possessed shorter teats. Negative strong genetic correlation also indicates that including one trait of those correlated traits in the selection program could affect negatively the population mean of the other trait, and therefore they should be carefully compromised in a selection program by giving them the appropriate weights. Information about the magnitude of genetic correlation between traits in animal breeding is important since it is related to every decision made with regards to the genetic selection program. Genetic selection on a particular trait (or traits) will have an indirect selection response to other traits which are associated genetically. This is critical specifically if the traits are correlated in an opposite way. Improving one trait might deteriorate the others.

The magnitude and direction of both genetic and phenotypic correlations among RLS, FA, UD, and TL reported in the literature are not consistent, for instance Toghiani (2011), Berry et al. (2004), and Němcová et al. (2011), where the same method as in the present study (the restricted maximum likelihood method) was used in the genetic analysis. Results of the present study were similar regardless of the direction, with those reported by Berry et al. (2004), Němcová et al. (2011), and Toghiani (2011) for some pairs of correlations, for example the genetic correlation of RLS–UD (-0.6 vs. -0.59; Toghiani, 2011), RLS–TL (0.101 vs. 0.09; Berry et al., 2004), or UD–TL (0.019 vs. -0.01; Zavadilová et al., 2011). These discrepancies could be attributed to the different population under study and how the traits were recorded. Generally, as is reported in the literature, linear traits were scored/typed, whereas in this project the traits were measured. Scoring/typing linear traits is cheaper than measuring the traits directly on animals. The recording is ideally done by certified classifiers when they are available so that the bias when typing/scoring the animals can be minimized. In the context of dairy cow breeding in Indonesia, linear traits have not been introduced into the breeding program; thus, the certified classifiers for typing linear traits are not yet available and hence the traits were measured instead of scored/typed.

## 4 Conclusions

Some conclusions can be drawn from the present study. Rear legs set, foot angle, udder depth, and teat length are heritable traits. Rear legs set, foot angle, udder depth, and teat length are correlated and the genetic correlations are stronger than those of phenotypic ones. This suggests that rear legs set, foot angle, udder depth, and teat length could be included in the selection program. Including the studied traits in the selection program should be done with proper weights so that an optimum selection response can be achieved.

## Data Availability

The datasets generated during and/or analysed during the current study are not publicly available because they are part of the ongoing dissertation research project, but are available from the corresponding author on reasonable request.
